# *catena*-Poly[[[pentaaquadysprosium(III)]-μ-5-az­an­ium­ylisophthalato] dichloride monohydrate]

**DOI:** 10.1107/S241431462501020X

**Published:** 2025-11-28

**Authors:** Ahlem Linda Boulkedid, Mehdi Boutebdja, Rochdi Ghallab

**Affiliations:** a Environmental Molecular and Structural Chemistry Research Unit University of Constantine-1 25000 Constantine Algeria; b Laboratoire de technologie des materiaux avances Ecole Nationale Polytechnique de Constantine Algeria; University of Aberdeen, United Kingdom

**Keywords:** crystal structure, dysprosium(III), 5-amino­isophtalic acid, polymeric chain

## Abstract

In the title compound, {[Dy(C_8_H_6_NO_4_)(H_2_O)_5_]Cl_2_·H_2_O}_*n*_ the central Dy^III^ atom is nine-coordinated by four oxygen atoms from two symmetry-related chelating carboxyl­ate groups and five water mol­ecules, resulting in a distorted polyhedral geometry best described as ‘muffin’-type by continuous shape measure analysis. The bridging ligands generate infinite [10] chains and the structure is consol­idated by an extensive hydrogen-bonding network and weak π–π stacking inter­actions.

## Abstract 2

The title compound contains zigzag polymeric chains built up from nine-coordinated Dy^III^ centers and bridging 5-az­an­ium­yl­isophthalate ligands.

## Graphical abstract

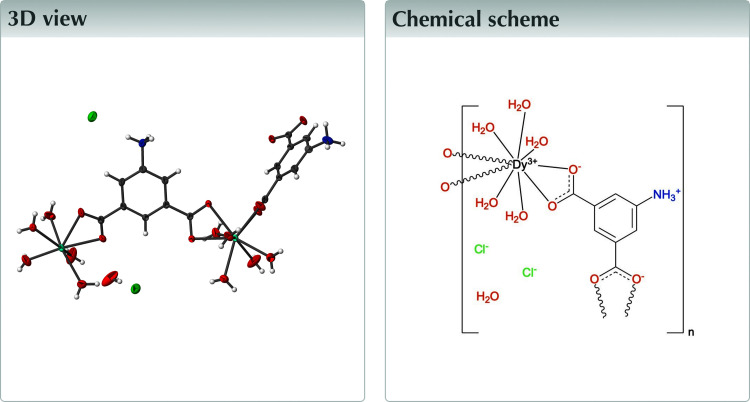

## Structure description

Single-crystal X-ray diffraction analysis reveals that the title compound, (**I**), crystallizes in the monoclinic space group *P*2_1_/*n*. The asymmetric unit consists of one Dy^3+^ ion, one 5-az­an­ium­yl­isophthalate ligand (C_8_H_6_NO_4_^−^ or AIP^−^), five coordinating water mol­ecules, two chloride anions and one water mol­ecule of crystallization (O6*W*). Both carboxyl­ate groups of the AIP ligand are deprotonated and the amino group is protonated.

**Figure 1:**
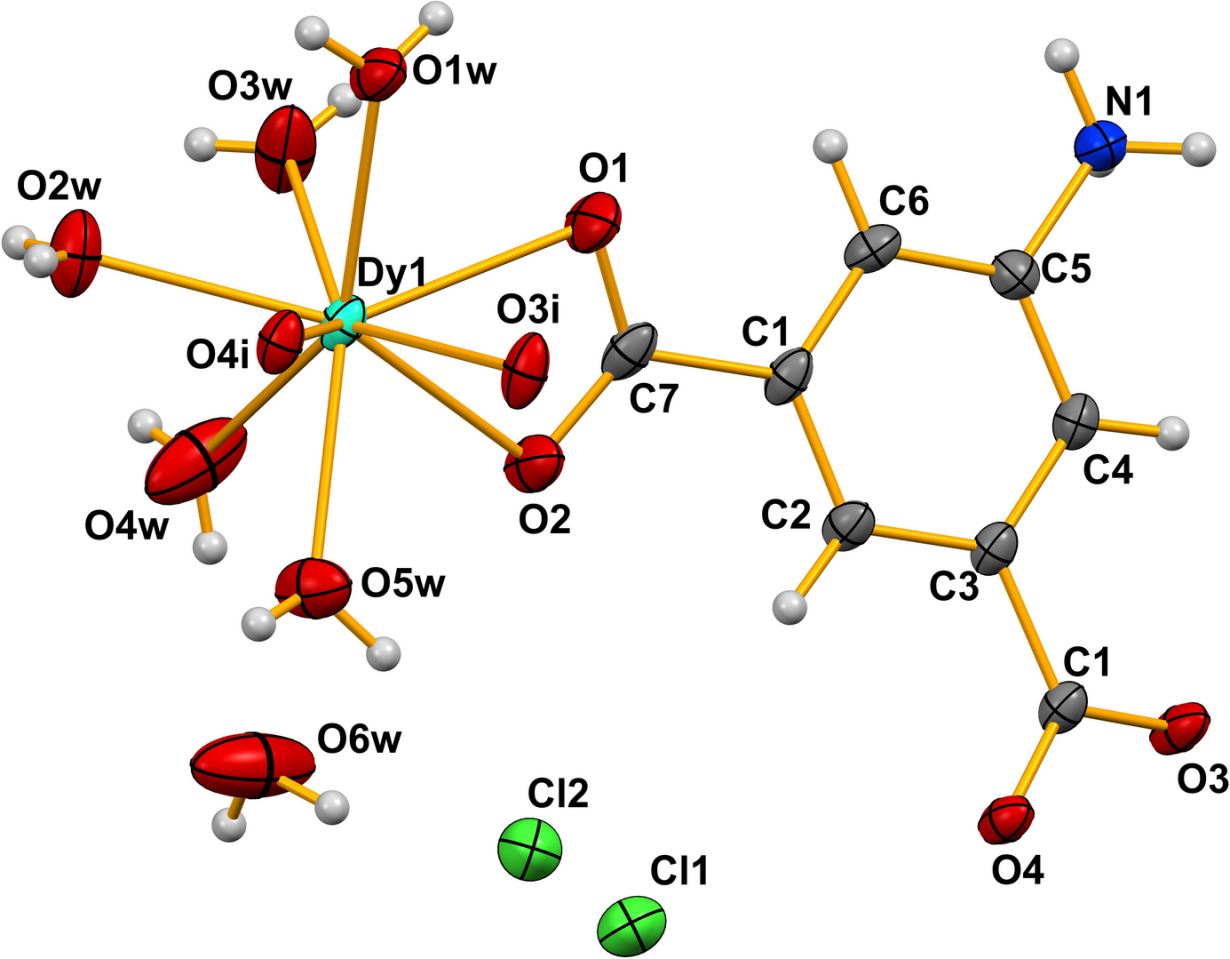
The asymmetric unit of (**I**) expanded to show the complete metal-ion coordination polyhedron with displacement ellipsoids for non-H atoms drawn at the 50% probability level. Symmetry code: (i) *x* − 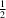, −*y* + 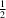, *z* − 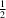.

**Figure 2:**
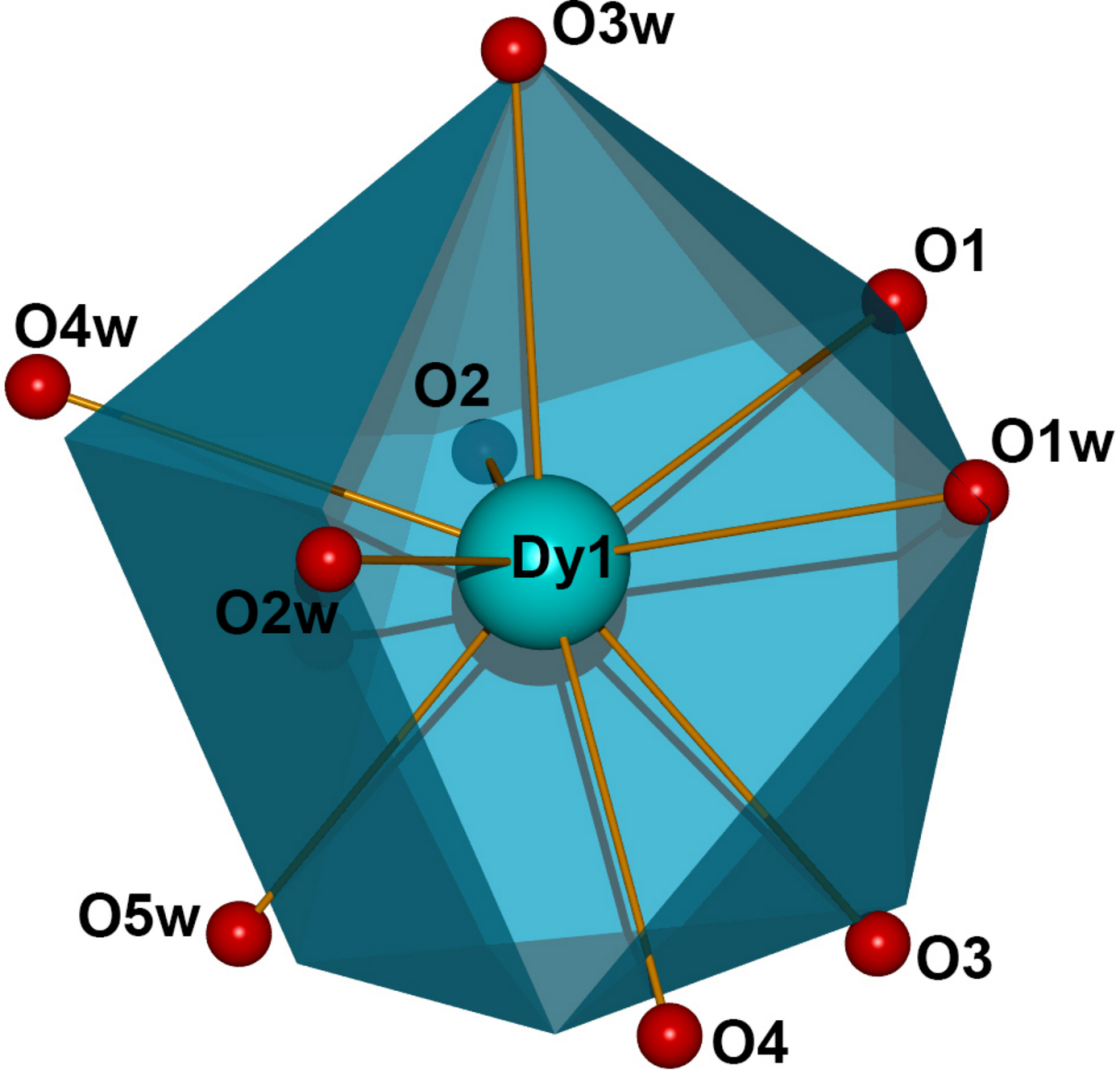
Distorted muffin-type coordination polyhedron of the nine-coordinate Dy1 atom in (**I**).

The metal ion in (**I**) exhibits a DyO_9_ coordination environment (Fig. 1 ▸): four oxygen atoms (O1 + O2 and O3 + O4) originate from symmetry-related chelating carboxyl­ate groups of two AIP^−^ ligands and the remaining atoms (O1*W*–O5*W*) arise from coordinating water mol­ecules. The Dy—O bond lengths range from 2.362 (3) to 2.4770 (19) Å (Table 1 ▸) and the O—Dy—O bond angles span the range 52.67 (6)° to 147.25 (9)°. An analysis of the DyO_9_ coordination polyhedron using the continuous shape measure (CShM) approach, as implemented in the *SHAPE* program (Casanova *et al.*, 2004 ▸), indicates that the DyO_9_ environment in (**I**) is inter­mediate between several ideal polyhedra, with the lowest deviation corresponding to a muffin-type geometry with *C*_s_ local symmetry (Table 2 ▸ and Fig. 2 ▸).

**Figure 3:**
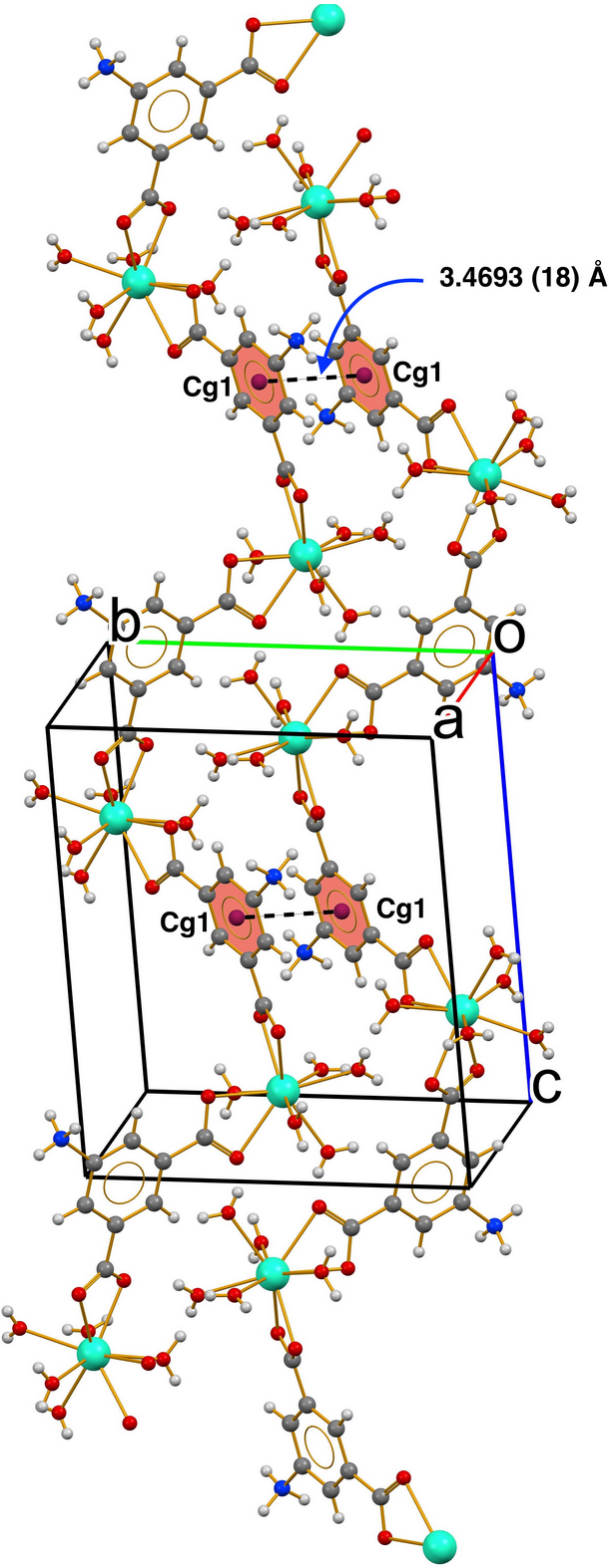
View of a fragment of an infinite [10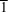] chain in (**I**) constructed by bridging AIP ligands and π–π stacking inter­actions represented as black dashed lines.

Each AIP^−^ ligand chelates two symmetry-equivalent Dy^3+^ ions *via* its carboxyl­ate groups, which leads to the formation of an infinite [10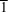] zigzag polymeric chain (Fig. 3 ▸). The shortest Dy⋯Dy separation within the chain is 9.8546 (5) Å while the shortest distance between chains is 6.2048 (4) Å. Otherwise, the geometrical parameters for (**I**) are consistent with reported values for similar compounds (Queirós *et al.*, 2021 ▸; Kariem *et al.*, 2017 ▸; Zhao *et al.*, 2012 ▸; Yan *et al.*, 2009 ▸; Ye *et al.*, 2008 ▸).

In the extended structure of (**I**), adjacent chains are cross-linked by an extensive hydrogen-bonding network featuring O—H⋯O, O—H⋯Cl, N—H⋯O and N—H⋯Cl inter­actions, some of which are bifurcated (Table 3 ▸) and slightly offset π–π stacking inter­actions between centrosymmetrically related phenyl rings [*Cg*1⋯*Cg*1 = 3.4693 (18) Å, slippage = 1.237 Å] also occur. Collectively, these inter­actions generate a three-dimensional supra­molecular network. For packing views, see Figs. S1–S3 in the supporting information.

## Synthesis and crystallization

A methano­lic solution of 5-amino­isophthalic acid (0.181 g, 1.00 mmol in 10 ml methanol) was added dropwise, under stirring, to a separate methano­lic solution of DyCl_3_·6H_2_O (0.094 g, 0.25 mmol). Upon stirring for several minutes, the reaction mixture yielded a white precipitate, which was collected by filtration and discarded. The colourless filtrate was allowed to evaporate slowly at room temperature over the course of one week, resulting in the formation of colorless blocks of (**I**), suitable for X-ray diffraction analysis.

## Refinement

Crystal data, data collection and structure refinement details are summarized in Table 4 ▸.

## Supplementary Material

Crystal structure: contains datablock(s) global, I. DOI: 10.1107/S241431462501020X/hb4543sup1.cif

Structure factors: contains datablock(s) I. DOI: 10.1107/S241431462501020X/hb4543Isup2.hkl

Packing views. DOI: 10.1107/S241431462501020X/hb4543sup3.docx

CCDC reference: 2502795

Additional supporting information:  crystallographic information; 3D view; checkCIF report

## Figures and Tables

**Table 1:** Selected geometric parameters (Å, °)

Dy1—O1	2.446 (2)	Dy1—O4*W*	2.449 (4)
Dy1—O1*W*	2.362 (3)	Dy1—O5*W*	2.367 (3)
Dy1—O2	2.407 (2)	Dy1—O3^i^	2.468 (2)
Dy1—O2*W*	2.396 (3)	Dy1—O4^i^	2.4770 (19)
Dy1—O3*W*	2.423 (2)		
			
O1—Dy1—O2	53.71 (8)	O3^i^—Dy1—O4^i^	52.67 (6)

Symmetry code: (i) 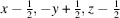.

**Table 2:** Analysis of the shapes of the coordination polyhedron of the Dy^3+^ ion performed by the *SHAPE* program

Shape	Deviation
Spherical-relaxed capped cube	9.13
Capped square anti­prism	2.47
Spherical capped square anti­prism	1.65
Tricapped trigonal prism	2.82
Spherical tricapped trigonal prism	1.62
Tridiminished icosa­hedron	11.27
Hula-hoop	10.29
Muffin	1.55

**Table 3:** Hydrogen-bond geometry (Å, °)

*D*—H⋯*A*	*D*—H	H⋯*A*	*D*⋯*A*	*D*—H⋯*A*
N1—H1*A*⋯Cl2^ii^	0.90 (5)	2.24 (5)	3.127 (4)	166 (4)
N1—H1*B*⋯Cl1	0.86 (3)	2.29 (4)	3.141 (4)	171 (3)
N1—H1*C*⋯O2^iii^	0.94 (5)	1.97 (4)	2.870 (4)	158 (4)
O1*W*—H1*WA*⋯O4^iv^	0.77 (5)	1.95 (5)	2.718 (3)	175 (5)
O1*W*—H1*WB*⋯Cl2^iv^	0.77 (5)	2.36 (5)	3.097 (3)	162 (4)
O2*W*—H2*WA*⋯Cl1^v^	0.85 (6)	2.38 (6)	3.209 (3)	163 (4)
O2*W*—H2*WB*⋯O6*W*^vi^	0.69 (6)	2.57 (6)	3.128 (5)	141 (7)
O3*W*—H3*WA*⋯Cl1^v^	0.77 (5)	2.44 (5)	3.186 (3)	163 (4)
O3*W*—H3*WB*⋯O3^iii^	0.75 (5)	2.12 (5)	2.780 (3)	148 (4)
O4*W*—H4*WA*⋯O6*W*	0.77 (5)	1.95 (5)	2.721 (5)	178 (5)
O4*W*—H4*WB*⋯Cl2^vii^	0.75 (6)	2.81 (5)	3.261 (4)	122 (4)
O4*W*—H4*WB*⋯Cl1^v^	0.75 (6)	2.64 (6)	3.273 (4)	144 (5)
O5*W*—H5*WA*⋯Cl1^viii^	0.77 (5)	2.32 (5)	3.085 (3)	170 (4)
O5*W*—H5*WB*⋯Cl2	0.87 (6)	2.21 (6)	3.075 (3)	171 (5)
O6*W*—H6*WA*⋯O1*W*^ix^	0.85	2.52	3.039 (4)	120
O6*W*—H6*WA*⋯O3^x^	0.85	2.49	3.161 (4)	137
O6*W*—H6*WB*⋯Cl2	0.85	2.32	3.126 (4)	158

Symmetry codes: (ii) 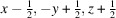; (iii) 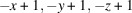; (iv) 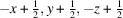; (v) 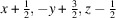; (vi) 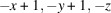; (vii) 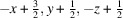; (viii) 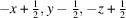; (ix) 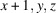; (x) 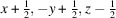.

**Table 4:** Experimental details

Crystal data	
Chemical formula	[Dy(C_8_H_6_NO_4_)(H_2_O)_5_]Cl_2_·H_2_O
*M*_r_	521.63
Crystal system, space group	Monoclinic, *P*2_1_/*n*
Temperature (K)	296
*a*, *b*, *c* (Å)	8.7274 (2), 11.9281 (3), 15.4697 (4)
β (°)	100.480 (1)
*V* (Å^3^)	1583.55 (7)
*Z*	4
Radiation type	Mo *K*α
μ (mm^−1^)	5.10
Crystal size (mm)	0.13 × 0.12 × 0.10

Data collection	
Diffractometer	Bruker APEXII CCD
Absorption correction	Multi-scan (*SADABS*; Krause *et al.*, 2015 ▸)
*T*_min_, *T*_max_	0.675, 0.747
No. of measured, independent and observed [*I* > 2σ(*I*)] reflections	16994, 4086, 3470
*R*_int_	0.042
(sin θ/λ)_max_ (Å^−1^)	0.676

Refinement	
*R*[*F*^2^ > 2σ(*F*^2^)], *wR*(*F*^2^), *S*	0.022, 0.052, 1.01
No. of reflections	4086
No. of parameters	266
H-atom treatment	H atoms treated by a mixture of independent and constrained refinement
Δρ_max_, Δρ_min_ (e Å^−3^)	0.86, −0.63

Computer programs: *APEX2* and *SAINT* (Bruker, 2013 ▸), *OLEX2.solve* (Bourhis *et al.*, 2015 ▸), *SHELXL2019/3* (Sheldrick, 2015 ▸), *OLEX2* (Dolomanov *et al.*, 2009 ▸) and *PLATON* (Spek, 2020 ▸).

## full crystallographic data

### catena-Poly[[[pentaaquadysprosium(III)]-µ-5-azaniumylisophthalato] dichloride monohydrate] Crystal data

**Table:**

[Dy(C_8_H_6_NO_4_)(H_2_O)_5_]Cl_2_·H_2_O	*F*(000) = 1012
*M**_r_* = 521.63	*D*_x_ = 2.188 Mg m^−^^3^
Monoclinic, *P*2_1_/*n*	Mo *K*α radiation, λ = 0.71073 Å
Hall symbol: -P 2yn	Cell parameters from 8978 reflections
*a* = 8.7274 (2) Å	θ = 2.7–34.0°
*b* = 11.9281 (3) Å	µ = 5.10 mm^−^^1^
*c* = 15.4697 (4) Å	*T* = 296 K
β = 100.480 (1)°	Block, colourless
*V* = 1583.55 (7) Å^3^	0.13 × 0.12 × 0.10 mm
*Z* = 4	

### catena-Poly[[[pentaaquadysprosium(III)]-µ-5-azaniumylisophthalato] dichloride monohydrate] Data collection

**Table:**

Bruker APEXII CCD diffractometer	3470 reflections with *I* > 2σ(*I*)
ω scans	*R*_int_ = 0.042
Absorption correction: multi-scan (SADABS; Krause *et al.*, 2015)	θ_max_ = 28.7°, θ_min_ = 2.2°
*T*_min_ = 0.675, *T*_max_ = 0.747	*h* = −11→11
16994 measured reflections	*k* = −13→16
4086 independent reflections	*l* = −20→20

### catena-Poly[[[pentaaquadysprosium(III)]-µ-5-azaniumylisophthalato] dichloride monohydrate] Refinement

**Table:**

Refinement on *F*^2^	0 restraints
Least-squares matrix: full	Hydrogen site location: difference Fourier map
*R*[*F*^2^ > 2σ(*F*^2^)] = 0.022	H atoms treated by a mixture of independent and constrained refinement
*wR*(*F*^2^) = 0.052	W = 1/[Σ^2^(*F*O^2^) + (0.0152*P*)^2^ + 1.8767*P*] WHERE *P* = (*F*O^2^ + 2*F*C^2^)/3
*S* = 1.01	(Δ/σ)_max_ < 0.001
4086 reflections	Δρ_max_ = 0.86 e Å^−^^3^
266 parameters	Δρ_min_ = −0.63 e Å^−^^3^

### catena-Poly[[[pentaaquadysprosium(III)]-µ-5-azaniumylisophthalato] dichloride monohydrate] Special details

**Table:**

Geometry. Bond distances, angles etc. have been calculated using the rounded fractional coordinates. All su's are estimated from the variances of the (full) variance-covariance matrix. The cell esds are taken into account in the estimation of distances, angles and torsion angles

### catena-Poly[[[pentaaquadysprosium(III)]-µ-5-azaniumylisophthalato] dichloride monohydrate] Fractional atomic coordinates and isotropic or equivalent isotropic displacement parameters (Å^2^)

**Table:**

	*x*	*y*	*z*	*U*_iso_*/*U*_eq_	
Dy1	0.29223 (2)	0.48158 (2)	0.14719 (2)	0.0189 (1)	
Cl1	0.02552 (10)	0.70815 (8)	0.56158 (6)	0.0377 (3)	
Cl2	0.64436 (11)	0.17687 (9)	0.24575 (6)	0.0430 (3)	
O1	0.2208 (3)	0.4945 (2)	0.29260 (14)	0.0313 (7)	
O1W	0.0349 (3)	0.5510 (2)	0.12853 (16)	0.0277 (7)	
O2	0.4427 (3)	0.4136 (2)	0.28306 (12)	0.0295 (7)	
O2W	0.2900 (3)	0.5851 (3)	0.01427 (17)	0.0365 (9)	
O3	0.6343 (3)	0.18700 (19)	0.66052 (12)	0.0262 (6)	
O3W	0.3298 (4)	0.6758 (2)	0.19189 (18)	0.0424 (9)	
O4	0.6313 (2)	0.12560 (18)	0.52657 (12)	0.0216 (6)	
O4W	0.5608 (4)	0.5387 (3)	0.1444 (3)	0.0608 (13)	
O5W	0.4398 (3)	0.3280 (2)	0.11052 (18)	0.0356 (8)	
N1	0.2551 (4)	0.5119 (3)	0.63105 (19)	0.0283 (9)	
C1	0.3633 (3)	0.4088 (2)	0.42259 (16)	0.0197 (8)	
C2	0.4623 (3)	0.3207 (3)	0.45410 (17)	0.0202 (8)	
C3	0.4866 (3)	0.2926 (2)	0.54279 (17)	0.0188 (8)	
C4	0.4162 (3)	0.3562 (3)	0.59983 (18)	0.0206 (8)	
C5	0.3188 (3)	0.4431 (3)	0.56745 (18)	0.0205 (8)	
C6	0.2882 (3)	0.4697 (3)	0.47899 (18)	0.0205 (8)	
C7	0.3396 (4)	0.4414 (3)	0.32713 (17)	0.0225 (8)	
C8	0.5899 (3)	0.1970 (2)	0.57829 (17)	0.0193 (8)	
O6W	0.8027 (3)	0.3937 (3)	0.1901 (3)	0.0748 (16)	
H1A	0.208 (5)	0.465 (4)	0.664 (3)	0.045 (12)*	
H1B	0.188 (4)	0.561 (3)	0.607 (2)	0.026 (9)*	
H1C	0.339 (5)	0.550 (4)	0.666 (3)	0.042 (11)*	
H2	0.508 (4)	0.281 (3)	0.414 (2)	0.024 (8)*	
H1WA	−0.007 (5)	0.574 (4)	0.084 (3)	0.045 (12)*	
H4	0.431 (3)	0.340 (3)	0.659 (2)	0.021 (8)*	
H1WB	0.007 (5)	0.578 (4)	0.168 (3)	0.048 (13)*	
H4WA	0.631 (5)	0.499 (4)	0.157 (3)	0.034 (12)*	
H6	0.223 (5)	0.532 (3)	0.458 (3)	0.040 (11)*	
H3WA	0.368 (5)	0.716 (4)	0.163 (3)	0.057 (15)*	
H3WB	0.309 (5)	0.700 (4)	0.233 (3)	0.058 (15)*	
H4WB	0.590 (6)	0.597 (5)	0.141 (3)	0.070 (18)*	
H5WA	0.437 (5)	0.301 (4)	0.065 (3)	0.058 (15)*	
H5WB	0.490 (6)	0.287 (5)	0.153 (4)	0.09 (2)*	
H2WA	0.351 (6)	0.641 (5)	0.015 (3)	0.09 (2)*	
H2WB	0.255 (7)	0.562 (6)	−0.025 (4)	0.09 (2)*	
H6WA	0.90093	0.40173	0.20341	0.1120*	
H6WB	0.78481	0.33146	0.21305	0.1120*	

### catena-Poly[[[pentaaquadysprosium(III)]-µ-5-azaniumylisophthalato] dichloride monohydrate] Atomic displacement parameters (Å^2^)

**Table:**

	*U*^11^	*U*^22^	*U*^33^	*U*^12^	*U*^13^	*U*^23^
Dy1	0.0253 (1)	0.0155 (1)	0.0139 (1)	−0.0019 (1)	−0.0019 (1)	0.0003 (1)
Cl1	0.0421 (5)	0.0308 (5)	0.0412 (4)	0.0098 (4)	0.0102 (4)	0.0073 (4)
Cl2	0.0492 (5)	0.0431 (6)	0.0411 (5)	0.0161 (4)	0.0199 (4)	0.0112 (4)
O1	0.0359 (12)	0.0381 (15)	0.0179 (10)	0.0113 (10)	−0.0005 (9)	0.0068 (9)
O1W	0.0351 (12)	0.0284 (14)	0.0184 (11)	0.0103 (10)	0.0017 (9)	0.0028 (9)
O2	0.0401 (12)	0.0321 (14)	0.0146 (9)	0.0116 (10)	0.0005 (8)	−0.0006 (8)
O2W	0.0479 (15)	0.0388 (17)	0.0216 (12)	−0.0139 (13)	0.0031 (11)	0.0039 (11)
O3	0.0396 (12)	0.0226 (12)	0.0135 (9)	0.0113 (10)	−0.0025 (8)	−0.0010 (8)
O3W	0.080 (2)	0.0229 (15)	0.0264 (13)	−0.0117 (14)	0.0156 (13)	−0.0065 (11)
O4	0.0292 (10)	0.0184 (11)	0.0153 (9)	0.0058 (9)	−0.0009 (8)	−0.0009 (8)
O4W	0.0394 (17)	0.034 (2)	0.099 (3)	−0.0118 (15)	−0.0143 (16)	0.0201 (18)
O5W	0.0393 (14)	0.0370 (16)	0.0275 (13)	0.0097 (11)	−0.0022 (11)	−0.0086 (11)
N1	0.0331 (14)	0.0288 (17)	0.0247 (13)	0.0124 (13)	0.0099 (11)	0.0066 (11)
C1	0.0273 (14)	0.0151 (15)	0.0143 (12)	−0.0024 (11)	−0.0027 (10)	0.0011 (10)
C2	0.0289 (15)	0.0149 (15)	0.0154 (13)	0.0018 (12)	0.0006 (11)	−0.0013 (10)
C3	0.0233 (13)	0.0150 (15)	0.0170 (12)	−0.0010 (11)	0.0009 (10)	0.0019 (10)
C4	0.0238 (14)	0.0203 (16)	0.0174 (13)	−0.0007 (11)	0.0031 (10)	0.0040 (11)
C5	0.0199 (13)	0.0220 (16)	0.0201 (13)	0.0013 (11)	0.0053 (10)	0.0003 (11)
C6	0.0213 (13)	0.0164 (16)	0.0216 (13)	0.0026 (11)	−0.0019 (10)	0.0025 (11)
C7	0.0330 (15)	0.0125 (15)	0.0188 (13)	−0.0014 (12)	−0.0035 (11)	0.0001 (11)
C8	0.0239 (14)	0.0150 (15)	0.0176 (12)	0.0000 (11)	0.0004 (10)	0.0015 (10)
O6W	0.0356 (15)	0.035 (2)	0.156 (4)	0.0001 (14)	0.023 (2)	−0.004 (2)

### catena-Poly[[[pentaaquadysprosium(III)]-µ-5-azaniumylisophthalato] dichloride monohydrate] Geometric parameters (Å, º)

**Table:**

Dy1—O1	2.446 (2)	O4W—H4WB	0.75 (6)
Dy1—O1W	2.362 (3)	O4W—H4WA	0.77 (5)
Dy1—O2	2.407 (2)	O5W—H5WA	0.77 (5)
Dy1—O2W	2.396 (3)	O5W—H5WB	0.87 (6)
Dy1—O3W	2.423 (2)	N1—H1A	0.90 (5)
Dy1—O4W	2.449 (4)	N1—H1B	0.86 (3)
Dy1—O5W	2.367 (3)	N1—H1C	0.94 (5)
Dy1—O3^i^	2.468 (2)	C1—C6	1.388 (4)
Dy1—O4^i^	2.4770 (19)	C1—C7	1.505 (4)
O1—C7	1.249 (4)	C1—C2	1.391 (4)
O2—C7	1.268 (4)	C2—C3	1.391 (4)
O3—C8	1.266 (3)	C3—C8	1.495 (4)
O4—C8	1.265 (3)	C3—C4	1.389 (4)
N1—C5	1.466 (4)	C4—C5	1.376 (5)
O1W—H1WA	0.77 (5)	C5—C6	1.383 (4)
O1W—H1WB	0.77 (5)	C2—H2	0.93 (3)
O2W—H2WA	0.85 (6)	C4—H4	0.92 (3)
O2W—H2WB	0.69 (6)	C6—H6	0.96 (4)
O3W—H3WB	0.75 (5)	O6W—H6WA	0.8500
O3W—H3WA	0.77 (5)	O6W—H6WB	0.8500
			
O1—Dy1—O1W	72.21 (8)	Dy1—O2W—H2WB	119 (6)
O1—Dy1—O2	53.71 (8)	H2WA—O2W—H2WB	120 (7)
O1—Dy1—O2W	142.49 (10)	Dy1—O2W—H2WA	119 (3)
O1—Dy1—O3W	73.81 (9)	Dy1—O3W—H3WA	119 (3)
O1—Dy1—O4W	113.80 (12)	Dy1—O3W—H3WB	125 (4)
O1—Dy1—O5W	120.66 (9)	H3WA—O3W—H3WB	117 (5)
O1—Dy1—O3^i^	74.62 (7)	Dy1—O4W—H4WA	123 (3)
O1—Dy1—O4^i^	121.39 (7)	H4WA—O4W—H4WB	109 (5)
O1W—Dy1—O2	125.48 (8)	Dy1—O4W—H4WB	127 (4)
O1W—Dy1—O2W	81.68 (9)	H5WA—O5W—H5WB	113 (5)
O1W—Dy1—O3W	77.05 (10)	Dy1—O5W—H5WA	128 (3)
O1W—Dy1—O4W	142.40 (10)	Dy1—O5W—H5WB	118 (4)
O1W—Dy1—O5W	142.09 (9)	C6—C1—C7	119.0 (2)
O1W—Dy1—O3^i^	76.27 (8)	C2—C1—C6	120.6 (2)
O1W—Dy1—O4^i^	72.10 (7)	C2—C1—C7	120.3 (2)
O2—Dy1—O2W	147.25 (9)	C5—N1—H1B	114 (2)
O2—Dy1—O3W	92.80 (9)	C5—N1—H1C	108 (3)
O2—Dy1—O4W	74.77 (12)	H1A—N1—H1B	109 (4)
O2—Dy1—O5W	73.40 (9)	H1A—N1—H1C	111 (4)
O2—Dy1—O3^i^	82.93 (8)	H1B—N1—H1C	108 (4)
O2—Dy1—O4^i^	128.93 (7)	C5—N1—H1A	107 (3)
O2W—Dy1—O3W	74.44 (10)	C1—C2—C3	120.1 (3)
O2W—Dy1—O4W	72.53 (12)	C2—C3—C4	119.3 (3)
O2W—Dy1—O5W	96.49 (10)	C4—C3—C8	119.4 (2)
O2W—Dy1—O3^i^	125.05 (9)	C2—C3—C8	121.3 (2)
O2W—Dy1—O4^i^	72.81 (9)	C3—C4—C5	119.7 (3)
O3W—Dy1—O4W	70.05 (12)	C4—C5—C6	121.9 (3)
O3W—Dy1—O5W	139.22 (10)	N1—C5—C6	120.5 (3)
O3^i^—Dy1—O3W	143.51 (10)	N1—C5—C4	117.5 (3)
O3W—Dy1—O4^i^	137.50 (9)	C1—C6—C5	118.3 (3)
O4W—Dy1—O5W	69.32 (11)	O1—C7—C1	120.4 (3)
O3^i^—Dy1—O4W	141.18 (10)	O1—C7—O2	121.2 (3)
O4^i^—Dy1—O4W	122.94 (11)	O2—C7—C1	118.4 (3)
O3^i^—Dy1—O5W	74.09 (8)	O4—C8—C3	120.2 (2)
O4^i^—Dy1—O5W	71.26 (8)	O3—C8—O4	120.1 (2)
O3^i^—Dy1—O4^i^	52.67 (6)	O3—C8—C3	119.7 (2)
Dy1—O1—C7	91.76 (18)	C1—C2—H2	118 (2)
Dy1—O2—C7	93.07 (18)	C3—C2—H2	122 (2)
Dy1^ii^—O3—C8	93.80 (16)	C3—C4—H4	121 (2)
Dy1^ii^—O4—C8	93.39 (15)	C5—C4—H4	119 (2)
Dy1—O1W—H1WA	121 (3)	C1—C6—H6	121 (3)
Dy1—O1W—H1WB	119 (3)	C5—C6—H6	121 (3)
H1WA—O1W—H1WB	113 (5)	H6WA—O6W—H6WB	105.00
			
O1W—Dy1—O1—C7	169.7 (2)	O5W—Dy1—O4^i^—C8^i^	82.71 (16)
O2—Dy1—O1—C7	−2.97 (19)	Dy1—O1—C7—O2	5.3 (3)
O2W—Dy1—O1—C7	−142.2 (2)	Dy1—O1—C7—C1	−174.6 (3)
O3W—Dy1—O1—C7	−109.0 (2)	Dy1—O2—C7—O1	−5.4 (3)
O4W—Dy1—O1—C7	−50.1 (2)	Dy1—O2—C7—C1	174.5 (3)
O5W—Dy1—O1—C7	29.1 (2)	Dy1^ii^—O3—C8—O4	2.3 (3)
O3^i^—Dy1—O1—C7	89.5 (2)	Dy1^ii^—O3—C8—C3	−178.6 (2)
O4^i^—Dy1—O1—C7	114.8 (2)	Dy1^ii^—O4—C8—O3	−2.2 (3)
O1—Dy1—O2—C7	2.93 (19)	Dy1^ii^—O4—C8—C3	178.6 (2)
O1W—Dy1—O2—C7	−5.6 (2)	C6—C1—C2—C3	0.2 (4)
O2W—Dy1—O2—C7	135.6 (2)	C7—C1—C2—C3	178.5 (3)
O3W—Dy1—O2—C7	70.5 (2)	C2—C1—C6—C5	2.3 (4)
O4W—Dy1—O2—C7	138.9 (2)	C7—C1—C6—C5	−176.1 (3)
O5W—Dy1—O2—C7	−148.6 (2)	C2—C1—C7—O1	158.3 (3)
O3^i^—Dy1—O2—C7	−73.2 (2)	C2—C1—C7—O2	−21.6 (4)
O4^i^—Dy1—O2—C7	−100.8 (2)	C6—C1—C7—O1	−23.3 (4)
O1—Dy1—O3^i^—C8^i^	154.02 (19)	C6—C1—C7—O2	156.7 (3)
O1W—Dy1—O3^i^—C8^i^	79.03 (18)	C1—C2—C3—C4	−2.6 (4)
O2—Dy1—O3^i^—C8^i^	−151.75 (18)	C1—C2—C3—C8	178.8 (2)
O2W—Dy1—O3^i^—C8^i^	9.7 (2)	C2—C3—C4—C5	2.6 (4)
O3W—Dy1—O3^i^—C8^i^	123.13 (19)	C8—C3—C4—C5	−178.9 (3)
O4W—Dy1—O3^i^—C8^i^	−97.0 (2)	C2—C3—C8—O3	166.1 (3)
O5W—Dy1—O3^i^—C8^i^	−77.05 (18)	C2—C3—C8—O4	−14.8 (4)
O1—Dy1—O4^i^—C8^i^	−32.37 (18)	C4—C3—C8—O3	−12.5 (4)
O1W—Dy1—O4^i^—C8^i^	−87.40 (16)	C4—C3—C8—O4	166.7 (3)
O2—Dy1—O4^i^—C8^i^	34.15 (18)	C3—C4—C5—N1	−176.3 (3)
O2W—Dy1—O4^i^—C8^i^	−174.00 (17)	C3—C4—C5—C6	0.0 (5)
O3W—Dy1—O4^i^—C8^i^	−132.88 (18)	N1—C5—C6—C1	173.8 (3)
O4W—Dy1—O4^i^—C8^i^	131.09 (17)	C4—C5—C6—C1	−2.4 (5)

Symmetry codes: (i) *x*−1/2, −*y*+1/2, *z*−1/2; (ii) *x*+1/2, −*y*+1/2, *z*+1/2.

### catena-Poly[[[pentaaquadysprosium(III)]-µ-5-azaniumylisophthalato] dichloride monohydrate] Hydrogen-bond geometry (Å, º)

**Table:**

*D*—H···*A*	*D*—H	H···*A*	*D*···*A*	*D*—H···*A*
N1—H1*A*···Cl2^iii^	0.90 (5)	2.24 (5)	3.127 (4)	166 (4)
N1—H1*B*···Cl1	0.86 (3)	2.29 (4)	3.141 (4)	171 (3)
N1—H1*C*···O2^iv^	0.94 (5)	1.97 (4)	2.870 (4)	158 (4)
O1*W*—H1*WA*···O4^v^	0.77 (5)	1.95 (5)	2.718 (3)	175 (5)
O1*W*—H1*WB*···Cl2^v^	0.77 (5)	2.36 (5)	3.097 (3)	162 (4)
O2*W*—H2*WA*···Cl1^vi^	0.85 (6)	2.38 (6)	3.209 (3)	163 (4)
O2*W*—H2*WB*···O6*W*^vii^	0.69 (6)	2.57 (6)	3.128 (5)	141 (7)
O3*W*—H3*WA*···Cl1^vi^	0.77 (5)	2.44 (5)	3.186 (3)	163 (4)
O3*W*—H3*WB*···O3^iv^	0.75 (5)	2.12 (5)	2.780 (3)	148 (4)
O4*W*—H4*WA*···O6*W*	0.77 (5)	1.95 (5)	2.721 (5)	178 (5)
O4*W*—H4*WB*···Cl2^viii^	0.75 (6)	2.81 (5)	3.261 (4)	122 (4)
O4*W*—H4*WB*···Cl1^vi^	0.75 (6)	2.64 (6)	3.273 (4)	144 (5)
O5*W*—H5*WA*···Cl1^ix^	0.77 (5)	2.32 (5)	3.085 (3)	170 (4)
O5*W*—H5*WB*···Cl2	0.87 (6)	2.21 (6)	3.075 (3)	171 (5)
O6*W*—H6*WA*···O1*W*^x^	0.85	2.52	3.039 (4)	120
O6*W*—H6*WA*···O3^xi^	0.85	2.49	3.161 (4)	137
O6*W*—H6*WB*···Cl2	0.85	2.32	3.126 (4)	158

Symmetry codes: (iii) *x*−1/2, −*y*+1/2, *z*+1/2; (iv) −*x*+1, −*y*+1, −*z*+1; (v) −*x*+1/2, *y*+1/2, −*z*+1/2; (vi) *x*+1/2, −*y*+3/2, *z*−1/2; (vii) −*x*+1, −*y*+1, −*z*; (viii) −*x*+3/2, *y*+1/2, −*z*+1/2; (ix) −*x*+1/2, *y*−1/2, −*z*+1/2; (x) *x*+1, *y*, *z*; (xi) *x*+1/2, −*y*+1/2, *z*−1/2.
