# Supplementary material for: catena-Poly[[[pentaaquadysprosium(III)]-μ-5-az­an­ium­ylisophthalato] dichloride monohydrate]
Source: IUCrdata. 2025 Nov 28;10(Pt 11):x251020. doi: 10.1107/S241431462501020X (PMC12810320; doi:10.1107/S241431462501020X)
Supplement: Supplementary file 3 [file x-10-x251020-sup3.docx]

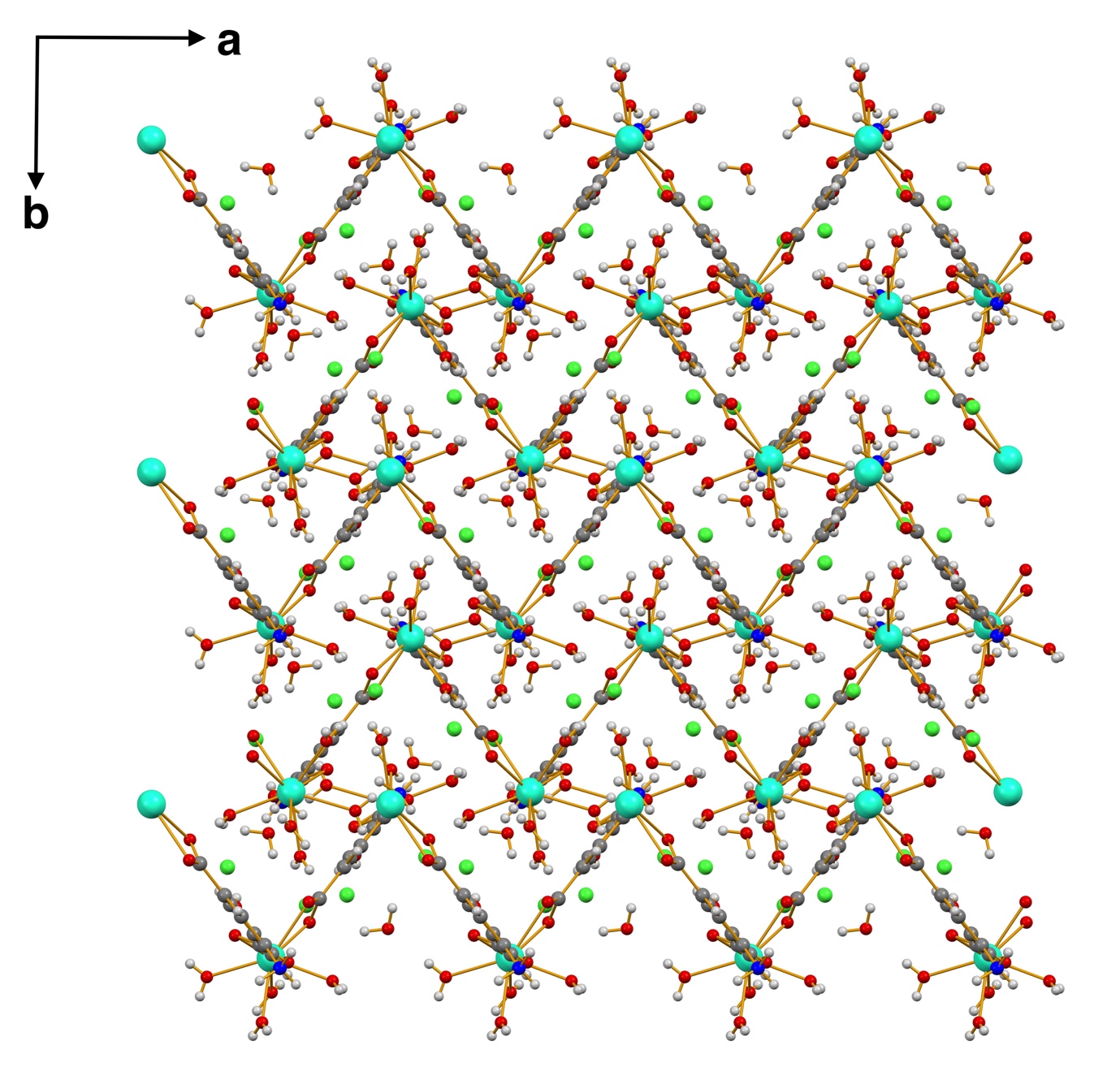


**Figure 4** View of the crystal packing arrangement along the [001] axis of the unit cell.


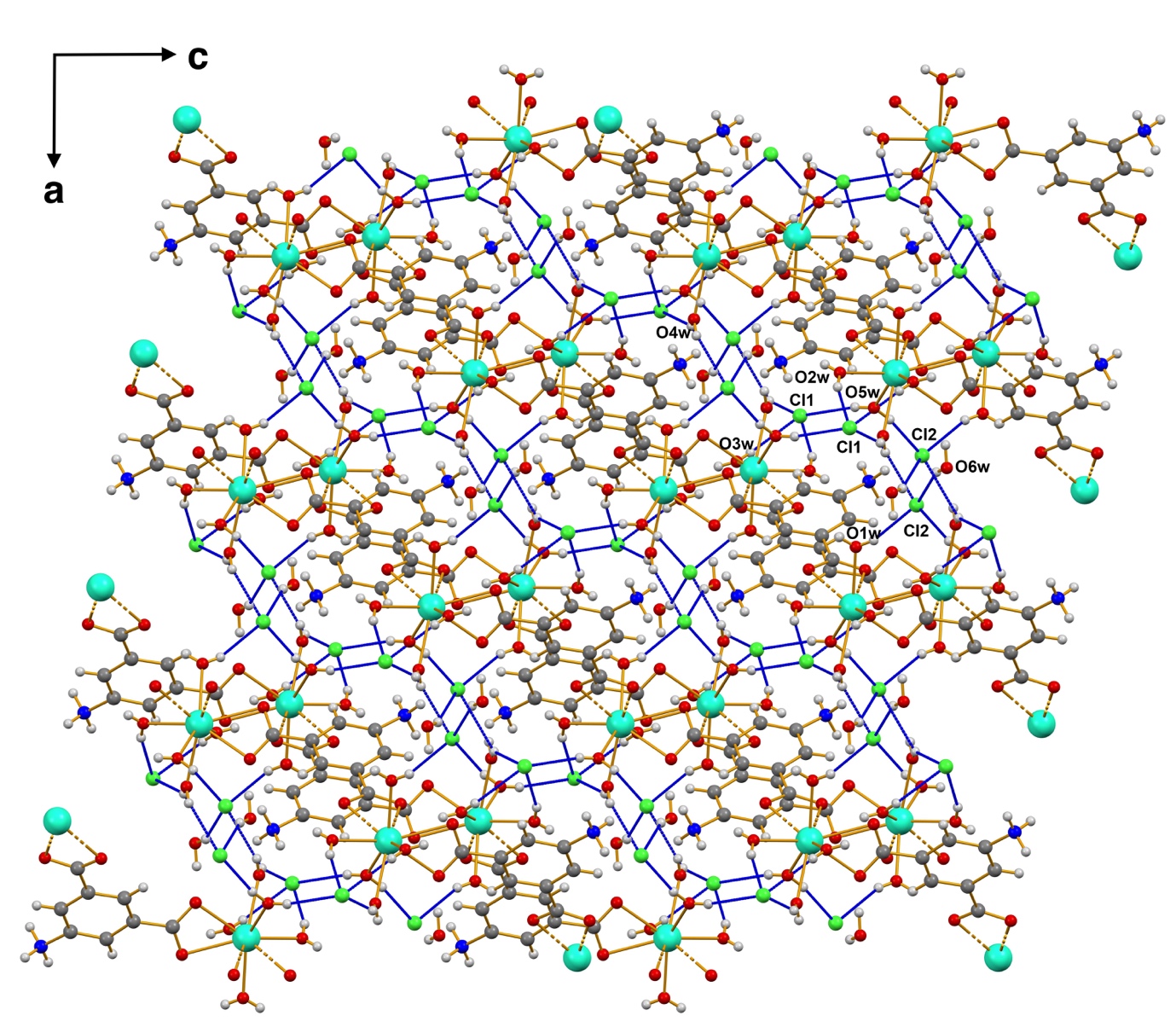


**Figure 5** Extended packing arrangement viewed in the [010] direction of the unit cell showed hydrogen bending interaction (blue line) between chloride anions and hydrogen atoms of coordinated water molecules.


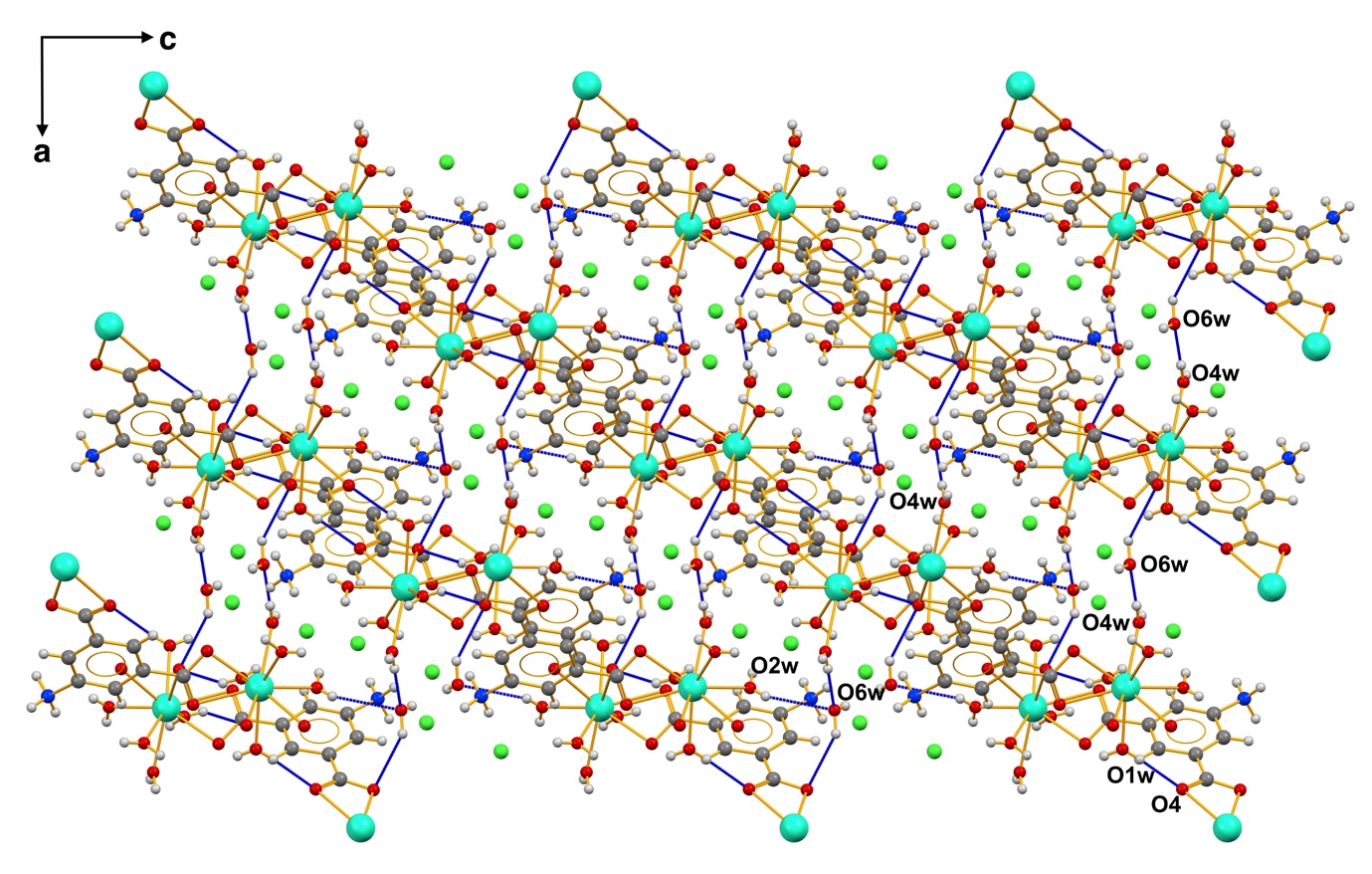


**Figure 6** Extended packing arrangement viewed in the [010] direction of the unit cell showed hydrogen bending interaction (blue line) between water molecules.
